# A Fellow-Led Quality Improvement Initiative to Increase Disease Activity Measurement in a Busy Academic Rheumatology Clinic

**DOI:** 10.7759/cureus.107509

**Published:** 2026-04-21

**Authors:** Morgan Lilly, Ruba Memon, Kamilla Guinn, Lauren Irwin, Kanika Monga

**Affiliations:** 1 Medicine, Texas A&M University School of Engineering Medicine, Houston, USA; 2 Rheumatology, Houston Methodist Hospital, Houston, USA; 3 Curriculum Development, Houston Methodist Hospital, Houston, USA; 4 Educational Research, Houston Methodist Hospital, Houston, USA

**Keywords:** academic rheumatology, artificial intelligence, clinical disease activity index (cdai), clinical workflow, electronic health record (ehr), quality improvement (qi), rheumatoid arthritis

## Abstract

Background: The preferred management of rheumatoid arthritis (RA), recommended by the American College of Rheumatology (ACR) and international organizations like the European Alliance of Associations for Rheumatology (EULAR), is the treat-to-target (T2T) strategy, due to its association with improved long-term patient outcomes. Successful implementation requires consistent assessment and documentation of disease activity, commonly using tools such as the Clinical Disease Activity Index (CDAI). However, variability in the use and documentation of disease activity measures exists in academic rheumatology settings.

Methods: This fellow-led quality improvement project utilized a Plan-Do-Study-Act (PDSA) framework to improve CDAI documentation in an academic rheumatology clinic. RA patient encounters were identified in both study periods using International Classification of Diseases, 10th Revision (ICD-10) code M06.9. Baseline (pre-intervention) data were collected retrospectively from July 1, 2022, to April 14, 2023. Following baseline assessment, a needs assessment survey was conducted, and an electronic health record (EHR)-integrated templated dot phrase was implemented as the intervention. Faculty and fellows also received email-based education regarding CDAI use and documentation. Post-intervention data were collected prospectively from April 17, 2023, to June 5, 2023. Pre- and post-intervention CDAI documentation rates were assessed through Epic EHR chart review at the encounter level, and the same patients were not necessarily included in both periods. Qualitative outcomes, including usability, workflow disruption, and perceived note bloat, were assessed through post-intervention provider surveys.

Results: Baseline data indicated 261 RA encounters, with only two containing documented CDAI scores. Following the intervention, CDAI was documented in 39 out of 54 RA encounters. Post-intervention surveys revealed that 100% of providers found the dot phrase useful, and 75% reported improved clinical behaviors related to disease activity assessment.

Conclusions: A simple EHR-based intervention, combined with provider engagement, significantly increased CDAI documentation, highlighting how fellow-led quality improvement initiatives may improve implementation of guideline-concordant care in rheumatology. These findings are consistent with prior research showing that EHR templates improve documentation efficiency and reportability and suggest that such interventions can support more effective adherence to ACR guidelines for rheumatoid arthritis management.

## Introduction

Rheumatoid arthritis (RA) is a chronic autoimmune disease characterized by synovial inflammation, joint damage, and systemic complications. The American College of Rheumatology (ACR), along with foreign associations like the European Alliance of Associations for Rheumatology (EULAR), recommended following a treat-to-target (T2T) approach for the management of RA, which requires assessing each patient’s disease severity. Several methods for assessing disease severity have been proposed since the 1990s, including the Disease Activity Score using 28 joint counts (DAS-28), Simplified Disease Activity Index (SDAI), Clinical Disease Activity Index (CDAI), Composite Activity Score Index (CASI), and Measure of Inflammatory Rheumatoid Arthritis (MOI-RA), and studies have compared and contrasted the benefits and limitations of each of these methods along with their differing ability to assess a patient’s RA disease severity. Historically, the DAS-28 was the most commonly used. However, it is less point-of-care friendly due to its reliance on and weighting of lab values, both ESR and CRP, to guide management decisions. Of the other disease severity assessments noted above, the CDAI score is the only one that does not require lab values, thereby increasing point-of-care friendliness. Instead, it incorporates aspects of the DAS-28, such as the 28 joint tender and swollen counts, with a patient global assessment score and a physician global assessment score in a simple algebraic formula. Although lab values are not required for the CDAI score, several studies have shown that it similarly predicts disease severity when compared to the DAS-28 and SDAI. Additionally, some studies have shown that the CDAI has a higher discriminative validity than the DAS-28 and a stricter threshold for indicating disease remission [1-3]. Together, these findings support CDAI as a valid, practical tool well-suited for routine use within the T2T framework. 

Because the T2T’s approach relies on frequent, accurate assessment of disease severity to guide treatment decisions, the consistent and correct implementation of validated tools such as the CDAI is essential for achieving optimal outcomes. The T2T approach of RA has been associated with improved long-term outcomes. However, successful implementation of the T2T approach relies on accurate disease severity measurement and reliable documentation over time, particularly in longitudinal clinic settings where multiple physicians or trainees may see the same patient [4,5]. Unfortunately, studies have shown that tools like CDAI are not routinely or consistently implemented in routine clinical practice.

We hypothesized that by equipping fellows and faculty with educational support coupled with an efficient electronic health record (EHR) documentation tool, CDAI utilization and accurate, consistent documentation of its score could be improved with minimal disruption to workflow. Specifically, we aimed to increase the proportion of RA encounters with documented CDAI scores in a busy academic rheumatology clinic through a fellow-led EHR-based quality improvement intervention.

## Materials and methods

Context

This was a single-center, pre-post interventional quality improvement (QI) study conducted at the Houston Methodist Rheumatology Clinic in the Texas Medical Center evaluating the impact of a fellow-led EHR-based intervention on documentation of the CDAI in outpatient RA encounters. “Fellow-led” refers to a QI initiative designed and implemented by rheumatology fellows under attending physician supervision, and “EHR-based” refers to an intervention embedded within the EHR to standardize clinical documentation.

**Figure 1:**
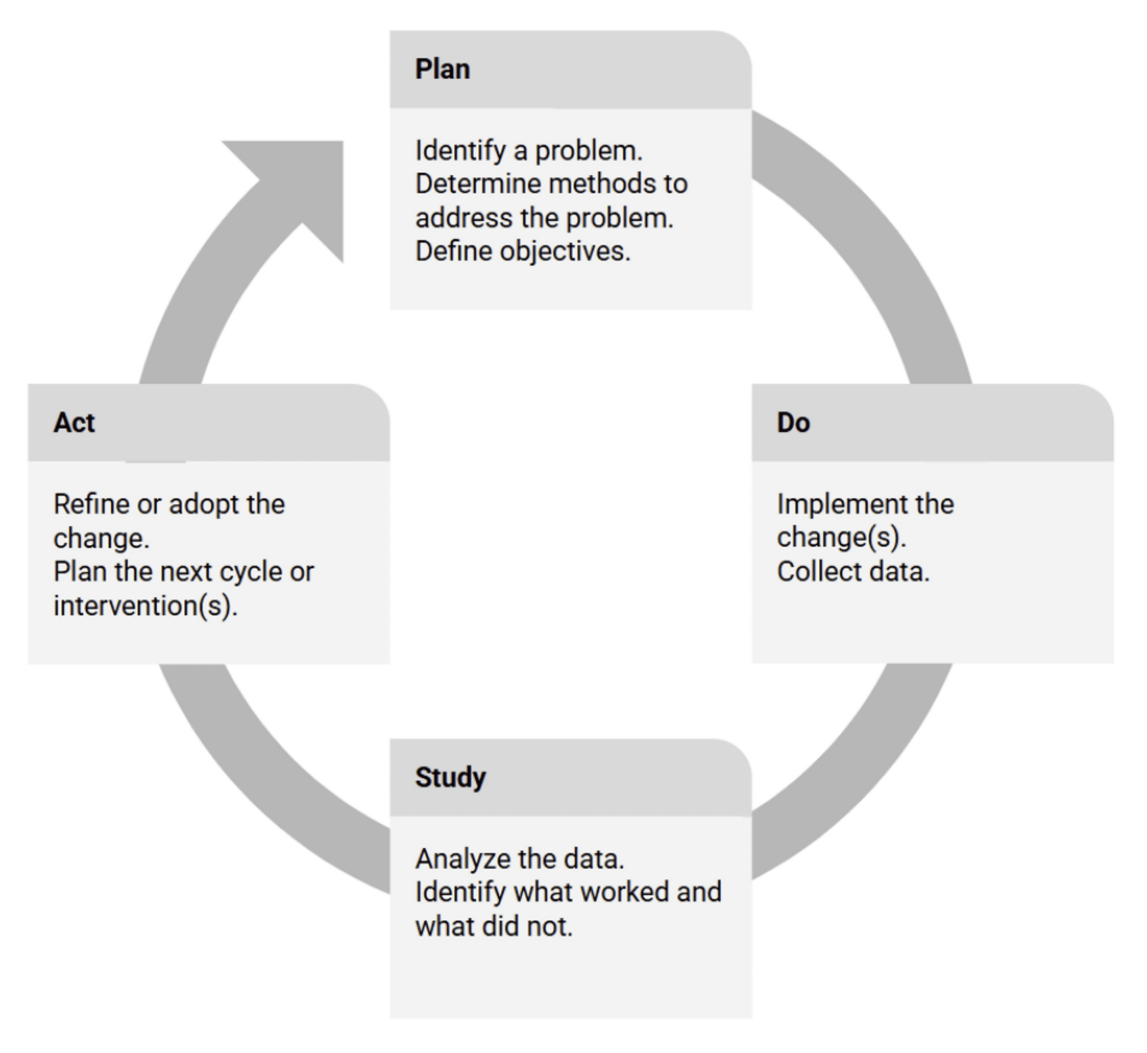
Plan-Do-Study-Act Quality Improvement Cycle Image created by the authors using Microsoft PowerPoint.

The study followed the Plan-Do-Study-Act (PDSA) cycle methodology for quality improvement. PDSA is a structured QI tool shown to facilitate iterative improvement by setting aims, defining measurements, implementing changes, testing interventions in real-world clinical settings, and reflecting on outcomes to guide refinement, as shown in Figure 1 [6].

The initiative combined retrospective and prospective EHR chart review with provider survey data to evaluate the impact of an EHR-based intervention designed to improve CDAI documentation. Within this framework, quantitative measures (EHR-derived CDAI documentation rates) and qualitative measures (provider survey responses) were integrated to assess both documentation outcomes and user experience.

The intervention was implemented within Epic, a widely used EHR system supporting clinical documentation and workflow integration. The clinic operates in a high-volume academic setting with approximately 18 patients per physician per day, of whom roughly 25% have RA. At baseline, no standardized CDAI documentation process existed, resulting in variability in its use and recording.

Study participants included rheumatology fellows and attending physicians documenting outpatient RA encounters. RA encounters were identified using the International Classification of Diseases, 10th Revision (ICD-10) code M06.9 within the EHR. Documentation outcomes were assessed at the encounter level.

The following describes details for the application of the PDSA cycle for this intervention.

Plan

To clarify the problem during the planning phase of the PDSA cycle, a pre-survey needs assessment was emailed to faculty and fellows at Houston Methodist Rheumatology, Texas Medical Center (Appendix I). Pre-survey results indicated that 60% (3/5) did not document disease activity in the EHR for patients with RA, while 20% occasionally documented scores (1/5). Reported barriers to using disease activity measures included time constraints during clinic visits and forgetting to collect a patient’s global score.

Do

Based on identified barriers, an EHR-integrated CDAI dot phrase was developed and implemented within Epic to standardize calculation and documentation of CDAI components. The dot phrase included tender joint count (28 joints), swollen joint count (28 joints), patient global assessment, and physician global assessment. An educational email was also sent to fellows and attending physicians describing the importance of disease activity measurement and providing instructions for use of the dot phrase. No mandatory training sessions were required to maintain a low-burden implementation strategy.

Study

Pre-intervention data were collected retrospectively through Epic EHR chart reviews to determine the baseline percentage of RA patients with documented CDAI scores between October 17, 2022, and April 14, 2023. RA patient encounters were identified using ICD-10 diagnostic codes for RA (M06.9) within the EHR. In this pre-post study design, the pre-intervention period served as the baseline comparator for evaluating changes following implementation of the EHR dot phrase described in the "Do" phase of the PDSA cycle. 

Following implementation, prospective data were collected from April 17, 2023, to June 5, 2023. The post-intervention period represented exposure to the EHR dot phrase, while the pre-intervention period served as the unexposed baseline. CDAI documentation was assessed at the encounter level, and the same individual patients were not necessarily included in both pre- and post-intervention periods. The difference in duration between study periods reflects real-world implementation constraints, including timing of intervention rollout and feasibility within the academic clinic schedule.

The primary outcome was the proportion of RA encounters with documented CDAI scores before and after implementation. Secondary qualitative measures were obtained through a post-intervention provider survey distributed to rheumatology fellows and attending physicians following implementation of the intervention (Appendix II). The survey included structured questions assessing usability, workflow integration, and perceived impact on clinical documentation and patient care. Survey responses were analyzed qualitatively using thematic review to identify recurring patterns in provider experiences. These included provider-reported ease of use, perceived workflow disruption, and concerns regarding documentation burden (“note bloat”), as well as perceived improvements in the ability to capture objective disease activity data and longitudinal assessment of patient outcomes.

Act

Based on provider feedback, iterative modifications were made to the dot phrase for future studies. One adjustment included adding a calculator that automatically summed components in the CDAI score. Another modification involved including the scoring cut-offs for remission, low, moderate, or high activity, embedded in the note templates for easier result interpretation. To prevent note bloat, the table size where the disease activity score is inserted was reduced. 

Ethical considerations

The project was reviewed by the Houston Methodist Institutional Review Board and determined to be exempt. 

## Results

A total of 261 RA patient encounters were identified during the pre-intervention period between July 1, 2022, and April 14, 2023. Among these encounters, only two contained complete CDAI documentation, indicating very low baseline utilization of disease activity scoring. 

After implementation of the EHR dot phrase and educational intervention, 54 RA encounters were recorded between April 17, 2023, and June 5, 2023. CDAI scores were documented in 39 of these encounters (72%), representing a substantial increase in the proportion of visits with recorded disease activity measurements, as shown in Table 1.

**Table 1:** Frequency of CDAI Documentation in Rheumatoid Arthritis Pre- and Post-Intervention CDAI: Clinical Disease Activity Index, RA: rheumatoid arthritis

	Pre-Intervention	Post-Intervention
Total RA Encounters (n)	261	54
Encounters with CDAI Documented (n)	2	39
Encounters with CDAI Documented (%)	0.77%	72.22%

**Figure 2:**
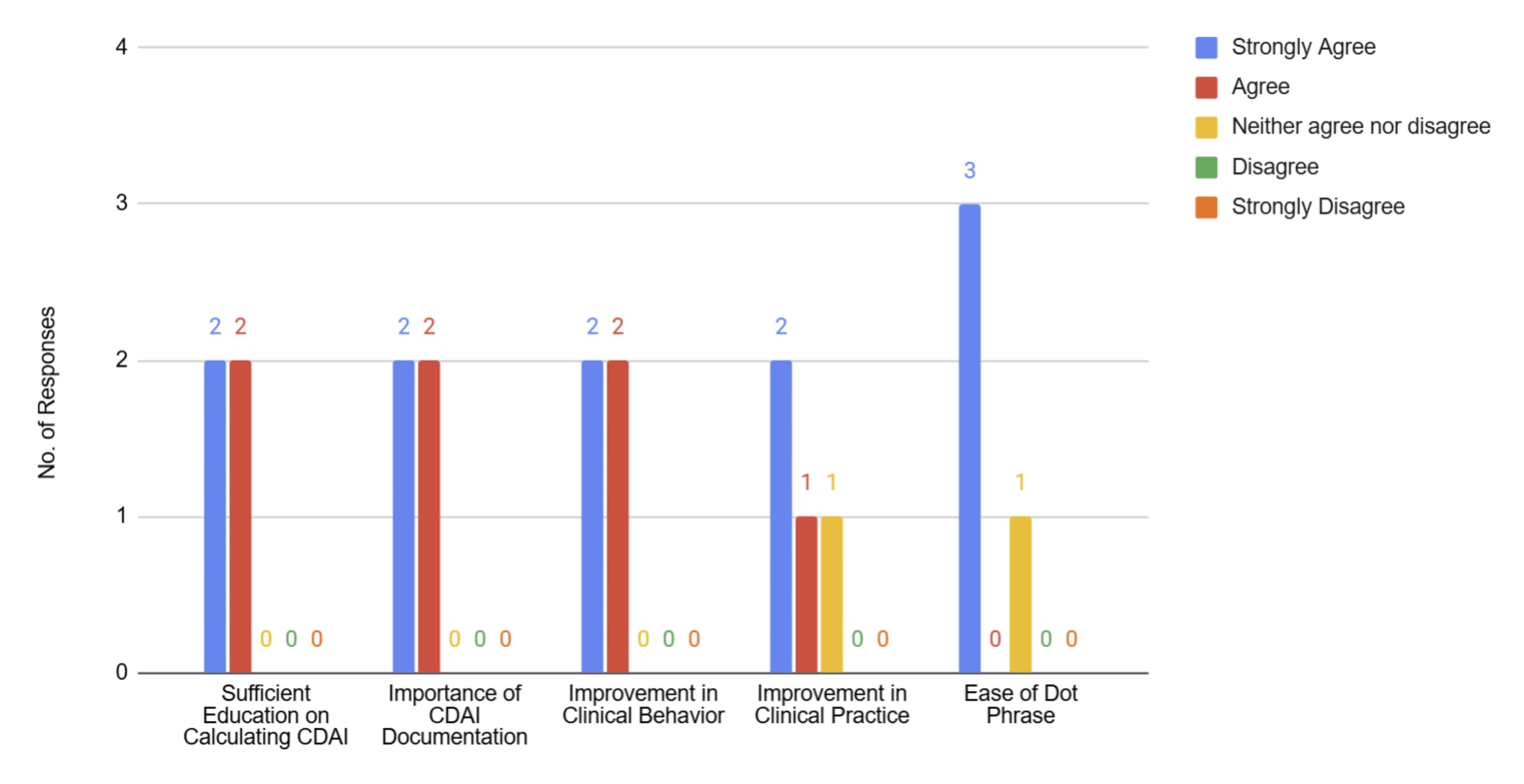
Participant Perceptions of Educational and EHR-Based Interventions CDAI: Clinical Disease Activity Index, EHR: electronic health record

Post-intervention survey results demonstrated positive perceptions of the intervention by rheumatology fellows and attendings. All physicians (4/4, 100%) strongly agreed or agreed that the educational email sufficiently explained how to calculate and document CDAI scores and reported an increased awareness of the clinical disease activity index assessment. Additionally, 75% (3/4) of participants strongly agreed or agreed that the dot phrase made documentation easier, and the same proportion reported that the intervention improved their clinical practice (Figure 2). Qualitative feedback indicated that physicians were also able to capture more objective data, leading to improved longitudinal assessment of patients’ disease activity and a clearer evaluation of treatment efficacy.

## Discussion

This study has identified and addressed the challenges faced by a busy academic rheumatology clinic in accurately calculating and documenting CDAI in patients with RA. Prior to the intervention, CDAI documentation was extremely limited despite guideline recommendations supporting routine disease activity measurement as part of the treat-to-target approach. The treat-to-target strategy is recommended by the ACR because RA is a chronic disease with intermittent remission and relapses, and its management should involve regular monitoring and alleviation of symptoms to achieve sustained low disease activity or remission [7]. Consistent and accurate documentation of disease activity is essential for long-term disease management, as longitudinal tracking of CDAI guides therapeutic decisions and treatment adjustments [8]. The CDAI provides a standardized method to monitor disease state over time, making it a valuable tool in routine clinical practice. Therefore this quality improvement initiative was completed to standardize the monitoring and management of patients with RA in a busy rheumatology clinic with multiple providers and to improve long-term patient care effectiveness. 

The implementation of a dot phrase in EHR proved to be effective in increasing the frequency of CDAI documentation for disease activity scoring. By integrating a dot phrase into clinicians’ workflows, this study addressed common barriers such as inconsistent documentation practices and time constraints, reducing the cognitive and administrative burden associated with calculating and entering CDAI scores. Similar to other health information technology interventions, structured documentation templates have been shown to improve documentation quality and process adherence in various clinical settings. For example, Gandrup et al. showed that health IT interventions increased documentation of RA disease activity scores in an academic rheumatology clinic [9]. Similarly, Bajaj et al. demonstrated that electronic reminders and documentation templates significantly increased capture of disease activity measures in RA care [10]. Interventions integrating clinical decision support tools and standardized templates have also been associated with improved adherence to guideline-recommended practices in chronic disease management [11]. These findings align with broader evidence that EHR-based documentation tools and prompts can improve capture of recommended clinical data [12]. 

By utilizing a mixed-methods research design, incorporating both quantitative and qualitative approaches, this study successfully evaluated the impact of the intervention. As mentioned above, quantitative results demonstrated a substantial increase in CDAI documentation. Qualitative feedback was an equally important component of this evaluation because provider perceptions of feasibility, usability, and integration strongly influence adoption and sustainability of clinical interventions. Qualitative measures are crucial in quality improvement initiatives because stakeholder buy-in significantly influences the sustainability and long-term adoption of interventions, a process that is influenced by factors such as solution adaptability, complexity, and cost impact [13,14]. Furthermore, clinician workflow burden, including documentation complexity, cognitive workload, and time constraints, contributes to underutilization of recommended tools and resources. This reinforces the importance of EHR design that supports clinical usability and reduces cognitive burden [15]. In this study, providers reported that the dot phrase was useful, increased awareness of disease activity assessment, and was perceived as feasible within routine workflows in the rheumatology clinic. Based on this positive feedback and provider engagement, we expect sustainability of the intervention over time, with the capability to modify or enhance the dot phrase as needed in the EHR to maintain relevance and usability. These findings underscore that interventions will be more successful and sustainable when perceived as practical and in alignment with clinical needs. 

Comparatively, this study’s findings align with other reports showing that EHR-based tools and workflow enhancements can increase documentation of RA disease activity scores. Gandrup et al. reported that multiple health IT interventions increased documentation of RA disease activity scores in an academic rheumatology clinic, which supports the reproducibility of this study’s approach [9]. More broadly, a systematic review on interventions to improve inpatient hospital documentation found that education and implementation of a new EHR reporting system were the most successful interventions [16,17]. Taken together, these results reinforce the value of leveraging EHR templates and provider education to support consistent assessment practices. 

In conclusion, the study provides valuable insights into improving the assessment and documentation of CDAI, leading to enhanced clinical skills and delivery of high-quality patient care in busy academic rheumatology clinics. The findings and recommendations of this study can contribute to ongoing efforts in optimizing disease activity measurement and ultimately improving outcomes for patients with RA. 

Future PDSA cycles will focus on sustaining improvements by incorporating clinic reminders and expanding education to include other members of the care team like medical assistants and new trainees. Expanding the intervention to other clinical sites and practice settings may also be considered to evaluate the generalizability and scalability of these interventions across various practice environments.

Limitations 

Several limitations should be considered. This single-center quality improvement study in an academic rheumatology clinic may limit generalizability, and reliance on a single electronic health record system (Epic) may reduce applicability to other settings. The follow-up period was relatively short, and the pre- and post-intervention periods differed in duration and RA encounter volume, reflecting real-world constraints; long-term sustainability was not assessed.

CDAI documentation was evaluated at the encounter level and may be subject to the Hawthorne effect, as providers were aware of the initiative. Although formal inter-rater validation was not performed, documentation in this academic setting typically involved both trainees and attending physicians, providing some degree of oversight and consistency.

Despite these limitations, the study’s focus on provider documentation practices supports the validity of the findings. CDAI documentation rates improved following the intervention, consistent across chart review and provider survey data.

## Conclusions

Fellow-led quality improvement efforts can effectively drive behavior change, improve adherence to evidence-based guidelines, and enhance clinical care delivery. Coupling fellow-led efforts with integration of an EHR dot phrase was associated with a significant increase in CDAI documentation rates in academic rheumatology clinics. Improving the routine measurement of disease activity supports the implementation of T2T strategies and may ultimately enhance patient outcomes. However, this study was not designed to assess patient outcomes, and conclusions are limited to documentation behavior change. Future efforts will focus on sustaining improvements through ongoing clinical reminders and workflow reinforcement strategies, as well as expanding implementation to other clinical sites.

## Appendices

Appendix I: Pre-Intervention Survey Questionnaire

1. How often do you assess disease activity in rheumatoid arthritis (RA) patients in your clinical practice?

Never, Only if insurance asks for it, At every visit 

2. Which disease activity scoring tool do you primarily use in your practice for assessing RA patients? 

None, Other (please specify), Clinical Disease Activity Index (CDAI)

3. How familiar are you with the components and calculation of the disease activity score used in your practice? 

N/A - I do not use disease activity scoring tools in my practice, Somewhat familiar, Very familiar

4. What factors do you consider when assessing disease activity in RA patients? (Select all that apply) 

Tender joint count, Swollen joint count, Patient global assessment of disease activity, Acute-phase reactants (ESR or CRP), Duration of morning stiffness

5. How confident are you in interpreting disease activity scores to guide treatment decisions in RA patients? 

N/A - I do not use disease activity scoring tools in my practice, Somewhat confident, Very confident

6. How often do you document disease activity scores in the electronic medical record (EMR) for RA patients? 

Rarely or never, Occasionally, Regularly

7. What challenges do you face when using disease activity scoring tools in your practice, if any? 

Open-ended question

8. How often do you receive feedback or education on the appropriate use of disease activity scoring tools in RA management? 

Rarely or never, Occasionally, Regularly

9. Do you feel that incorporating disease activity scoring tools has improved the management of RA patients in your practice?

Yes, N/A

10. What resources or support do you need to better utilize disease activity scoring tools in RA management? 

Open-ended question

Appendix II: Post-Intervention Survey Questionnaire 

1. What is your current job title? 

Teaching Faculty, Faculty, Trainee (Resident or Fellow), Other (free response)

2. The initial email sent on 4/14/2023 regarding CDAI score calculations was sufficient in teaching me how to calculate the score. 

Strongly agree, Agree, Neither agree nor disagree, Disagree, Strongly disagree

3. The initial email sent on 4/14/2023 regarding CDAI score calculation was sufficient in teaching me the importance of documenting disease activity scores. 

Strongly agree, Agree, Neither agree nor disagree, Disagree, Strongly disagree

4. This intervention has positively impacted my clinical behavior. 

Strongly agree, Agree, Neither agree nor disagree, Disagree, Strongly disagree

5. This intervention has positively impacted my clinical practice. 

Strongly agree, Agree, Neither agree nor disagree, Disagree, Strongly disagree

6. The CDAI dot phrase makes it easier to document the disease activity score. 

Strongly agree, Agree, Neither agree nor disagree, Disagree, Strongly disagree

7. How did your patient care change as a result of this intervention? 

Enter your answer 

8. Are you having any difficulties using the dot phrase? If so, please describe. 

Enter your answer

9. What is the biggest barrier that you face when it comes to measuring and documenting disease activity scores? 

Enter your answer

10. Please share any additional thoughts and comments regarding this project (optional). 

Enter your answer
